# Hyaluronic Acid Hydrogels Crosslinked in Physiological Conditions: Synthesis and Biomedical Applications

**DOI:** 10.3390/biomedicines9091113

**Published:** 2021-08-30

**Authors:** Luis Andrés Pérez, Rebeca Hernández, José María Alonso, Raúl Pérez-González, Virginia Sáez-Martínez

**Affiliations:** 1 Instituto de Ciencia y Tecnología de Polímeros (ICTP-CSIC), c/Juan de la Cierva, 3, 28006 Madrid, Spain; lperez@imasmed.com; 2 i+Med S. Coop. Parque Tecnológico de Álava, Albert Einstein 15, Nave 15, 01510 Vitoria-Gasteiz, Spain; jalonso@imasmed.com (J.M.A.); rperez@imasmed.com (R.P.-G.)

**Keywords:** hyaluronic acid, cross-linking, physiological conditions

## Abstract

Hyaluronic acid (HA) hydrogels display a wide variety of biomedical applications ranging from tissue engineering to drug vehiculization and controlled release. To date, most of the commercially available hyaluronic acid hydrogel formulations are produced under conditions that are not compatible with physiological ones. This review compiles the currently used approaches for the development of hyaluronic acid hydrogels under physiological/mild conditions. These methods include dynamic covalent processes such as boronic ester and Schiff-base formation and click chemistry mediated reactions such as thiol chemistry processes, azide-alkyne, or Diels Alder cycloaddition. Thermoreversible gelation of HA hydrogels at physiological temperature is also discussed. Finally, the most outstanding biomedical applications are indicated for each of the HA hydrogel generation approaches.

## 1. Introduction

Hyaluronic acid (HA) is a non-sulfated glycosaminoglycan composed of repeating units of the disaccharide β-1,4-D-glucuronic acid–β-1,3 N-acetyl-D-glucosamine. This polysaccharide is naturally found in the human body, especially in connective tissues, skin, and synovial joint fluids. Apart from its biocompatibility and bio-functionality, HA displays physicochemical properties, such as high-water retention and viscoelastic properties, which make it the candidate of choice for bio-applications in several fields of medicine. Hyaluronic acid is employed as viscosupplement for the treatment of osteoarthritis, it constitutes a treatment for dry eye disease, and it is employed as an ingredient in dermatological and cosmetic formulations for skin care. Nevertheless, HA presents poor mechanical properties and rapid degradation via oxidative species and enzymatic degradation which hinder its use for some bio-applications. For example, for the use of hyaluronic acid as temporary scaffolds for tissue engineering applications, it is necessary to adjust the rate of degradation to the rate of formation of the new tissue. To overcome these drawbacks, the HA chains can be cross-linked, either chemically or physically to form hydrogels. The physicochemical properties, stability, and half-life of the native HA can be improved by modifying its structure and forming a hydrogel. After the reaction, the HA hydrogels can maintain the biocompatibility and biodegradability that characterize the unmodified material [1,2].

In a simple way, HA crosslinking can be carried out in two ways: by directly adding a cross-linker and forming the three-dimensional (3D) network, or by pre-modifying the HA chains with functional groups liable to be crosslinked. The latter leads to the generation of active moieties that also add new functionalities to the hydrogel [3]. The disaccharide units of HA possess three sites that may undergo chemical modification: the carboxyl group, hydroxyl group, and N-acetyl group [4,5]. HA is mainly modified through its carboxyl group affording amide formation using coupling reagents such as 4-(4,6-dimethoxy-1,3,5-triazin-2-yl)-4-methyl-morpholinium chloride (DMTMM) or carbodiimide derivatives, such as 1-ethyl-3-(3-dimethylaminopropyl) carbodiimide (EDC) [6] along with activating groups as N-hydroxybenzotriazole (HOBt) [7], N-hydroxysuccinimide (NHS) [8], or N-hydroxysulfosuccinimide (sulfo-NHS) [9]. On the other hand, the hydroxyl group can undergo different reactions such as oxidation by sodium periodate (NaIO_4_) [10]; hemiacetal formation; ether formation through reagents as 1,4-butanediol diglycidyl ether (BDDE) [11,12] or divinyl sulfone (DVS) [13]; and esterification [14]. Finally, N-acetyl groups may react through deacetylation and amidation.

**Figure 1:**
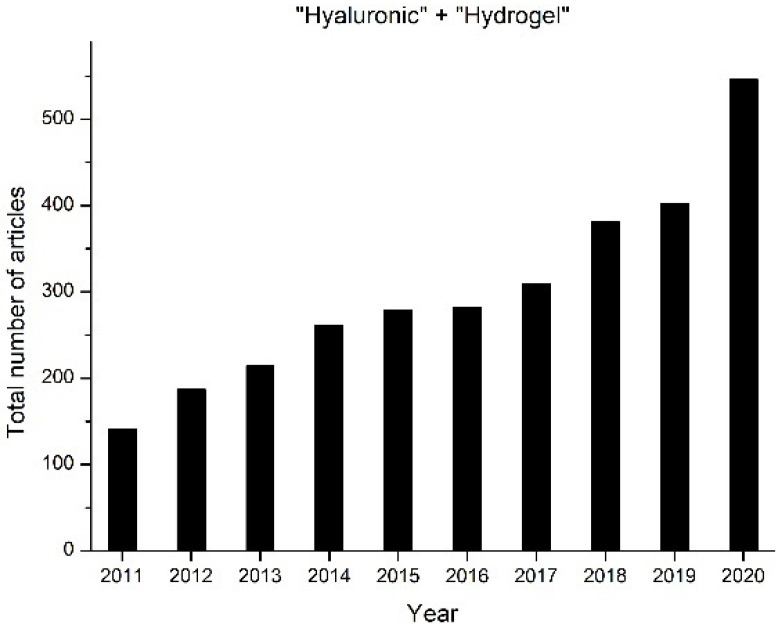
Number of papers that contain the terms ‘Hyaluronic’ and ‘Hydrogel’ (2011–2020) (Source: SCOPUS).

Over the last ten years, research interests in the development of hyaluronic acid hydrogels have increased exponentially as seen in Figure 1, which represents the number of papers with the words “Hyaluronic” and ”hydrogel” found in SCOPUS.

Divinyl sulfone (DVS) represents one of the most extended crosslinkers for the formation of hyaluronic acid hydrogels since the crosslinking process is simple, reproducible, and safe as it does not employ any organic solvents [15]. In a recent publication, some of us reported on the preparation and characterization of injectable hyaluronic hydrogels crosslinked with DVS at different HA: DVS weight ratios. The reaction between the hydroxyl groups present in the HA and DVS gives rise to ether formation and occurs at high pH values (0.25 M NaOH) and room temperature. An additional purification step was performed after the reaction (elimination of unreacted DVS) to obtain the final hydrogels. The hydrogels obtained at low HA:DVS weight ratios were non-cytotoxic and showed an excellent capacity to load antibiotics and anti-inflammatory agents [13]. In order to reach industrial production of hydrogels, a quality by design approach (QbD) must be adopted that predefines the properties of the targeted hydrogel. Such an approach is implemented by biomedical companies for the development of hyaluronic acid hydrogels that have reached the market in the form of injectable products [16]. Apart from DVS, glutaraldehyde, 1-Ethyl-3-(3-dimethyl aminopropyl) carbodiimide, or BDDE among others are traditionally employed for HA cross-linking. However, there might be biocompatibility issues with the resulting hydrogels. This is because some of them may possess in their structure unreacted cross-linkers which are known to be cytotoxic and can diffuse out of the hydrogel. In addition, the experimental conditions employed for the HA crosslinking reactions are often not compatible with cell culture conditions (pH 7.0–7.6, 37 °C) [17].

Another method of crosslinking for hyaluronic acid is ultraviolet (UV) photoinitiated crosslinking. Such an approach is often employed for the coupling of thiol groups, previously introduced within the HA backbone, to alkenes through a radical-mediated process. Thiol-ene photopolymerizations occur with any olefin bond, however, most of the HA hydrogels obtained in this way are produced through thiol- acrylate reactions. Hydrogels formed through thiol-acrylate photopolymerization display tunable mechanical properties by controlling factors that affect reaction kinetics (e.g., photopolymerization, light intensity, molecular weight, stoichiometry, and functionalities of monomers, temperature, chemical properties and concentration of initiators, and solvent choice) [18,19,20]. Although hydrogels formed by thiol-acrylate photopolymerization have found widespread applications in tissue engineering and regenerative medicine, photoinitiated crosslinking is also considered risky for some bio-applications as long irradiation periods required to improve the hydrogel mechanical properties might compromise cell viability [21].

This review is intended to provide an overview of different strategies to obtain hyaluronic acid-based hydrogels under physiological conditions and their biomedical applications. The review affords a brief outline of different chemical routes to crosslink HA at physiological pH and temperature, many of them involving click chemistry and the formation of dynamic (reversible) covalent bonds. A summary of the biomedical applications found in the literature is also provided. Different approaches for the preparation of thermoreversible hyaluronic hydrogels at physiological temperature including grafting or combination with thermoresponsive synthetic polymers (poly-N isopropylamide or pluronics) and natural polymers (gelatin or dextran among others) are reviewed as well.

## 2. Overview of HA Crosslinking Reactions Carried out at Physiological Conditions

The design of crosslinking strategies for hyaluronic acid hydrogel formation at physiological pH and T conditions, expands the range of biomedical applications for these materials, especially for those in which the gel formation occurs in the presence of living cells, proteins, or drugs. In recent years click chemistry, characterized by its high reactivity, selectivity, and yield, appears as the most promising strategy for the development of hydrogel under mild conditions. In addition, its unique bioorthogonality allows for gentle and efficient encapsulation of several bioactives onto the formed hydrogels [22,23,24].

Many of the current strategies for the formation of HA hydrogels at physiological conditions involve the formation of dynamic bonds which can break down and then reform with or without an external stimulus. The nature of the reversible bonds can be based on covalent or noncovalent (physical) interactions. Among non-covalent bonds, guest–host interactions are extensively employed in the formation of hyaluronic acid-based hydrogels under mild conditions [25,26,27]. However, these networks may have poor properties or low stability. To overcome these drawbacks and obtain the advantages that covalent crosslinking bond offers, hydrogels can incorporate two types of crosslinks: a prior physical crosslinking with fast gelation and self-healing capacity, and then a covalent crosslinking that provides stability and improves mechanical properties in the network [28,29].

**Figure 2:**
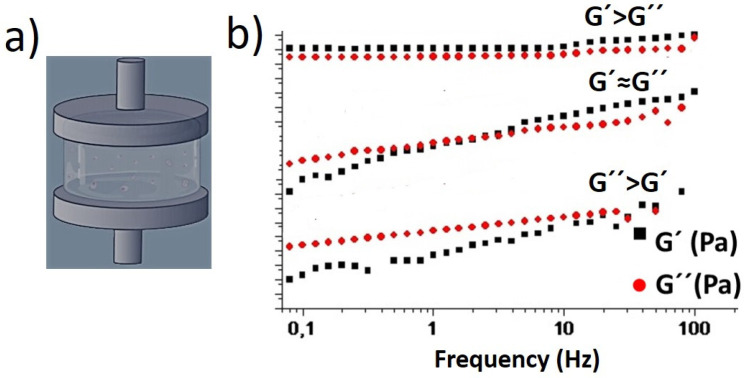
(**a**) Schematic representation of an experiment for the determination of G′ and G″ of hydrogels through shear rheometry. (**b**) Oscillatory frequency experiments performed on polymer hydrogels with different relaxation times starting from polymer solutions (with no crosslinks) to fully gel-like materials in which G′ > G″. Adapted with permission from [33].

Dynamic bonds endow the material with adaptability, self-healing capacity, stress relaxation, or shear thinning properties. Such features allow the material to flow and to be printed or injected under the shear forces and then, recover its macroscopic properties once the force/deformation has stopped. Nowadays, dynamic hydrogels attract a lot of attention due to their applications as injectable biomaterials, that allow the filling of irregularly shaped lesion sites with minimally invasive intervention [30,31,32]. The macroscopic properties of dynamic hydrogels are dependent on the network strand size and on the kinematic exchange of the dynamic bonds. A schematic representation of the experimental setup employed to measure the viscoelastic properties of hydrogels employing shear rheology is shown in Figure 2a. Figure 2b exemplifies the rheological behavior of a hydrogel in response to oscillatory frequency sweeps. In solution, in absence of crosslinker, the loss modulus, G″, is higher than the elastic modulus, G′ and they are both dependent on frequency. A fast-relaxing system shows viscoelastic behavior (G′ and G″ crossover within frequency ranges tested), whereas a slowly relaxing system shows a gel-like behavior (G′ > G″, within all frequency ranges tested). The determination of rheological properties for polymer hydrogels lies within the base for the development of their advanced applications. Specifically, for dynamic covalent hydrogels used in tissue engineering applications, the tuning of the viscoelastic properties of scaffolding hydrogels is employed as a strategy to control cell behavior in vitro [33,34].

In this section, the most used chemical routes employed to obtain hyaluronic acid hydrogels under physiological conditions are reviewed along with the biomedical applications from the resulting HA hydrogels.

### 2.1. Boronic-Ester Formation

**Figure 3:**
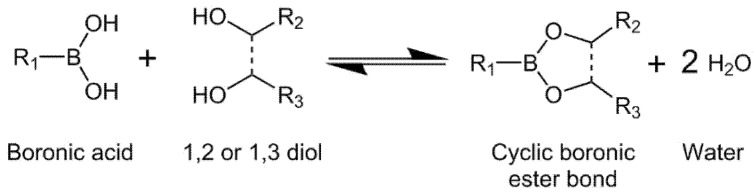
Formation of reversible boronic esters bond through reactions between boronic acids and diols.

Reversible boronic ester bonds can be formed by condensation reaction between boronic acids and cis-1, 2 or cis-1, 3 diols under mild conditions (Figure 3). These materials have gained importance due to their reversibility behavior under mild conditions as a function of the pH, which confers the network with self-healing capacity allowing their injectability [35,36]. Some key factors for the formation of the boronic ester bonds include the binding affinity of the boronic acid derivate towards the diol (Ka), their pKa, and the pH of the medium. The optimal pH for the formation of the ester bond can be found between the pKa values of boronic acid and the diol [37,38,39,40]. The estimation was proposed by “the charge rule”: “sum of the charges of the free esterifying species is equal to the charge of the ester” [41].

In this regard, dynamic hydrogels based in HA modified with phenylboronic acid (PBAs, pKa 8.8) groups and HA modified with maltose groups and gluconamide moieties (HA-GLU) were obtained under physiological conditions [42,43]. The gelation capability of HA-PBA was attributed to the interaction of negatively charged HA chains (carboxyl groups) with PBA lowering its pKa. The hydrogels showed self-healing ability and reversibility tailored by pH changes or by the addition of free glucose to the system [44]. Introducing an electron-withdrawing group or modifying the structure of the substituents in PBA may vary and reduce its pKa, enabling the reaction with hydroxyl groups at lower pH (including physiological conditions). It has been found that changes to the chemical structure of the boronic acid species (i.e., differences in the ortho-substitution in the PBA structure) result in differences in the reaction conditions for the formation of the gel which, in turn, influences the viscoelastic properties of the resulting hydrogels [45,46].

Besides the formation of hyaluronic-based macrogels, the generation of hyaluronic acid nanogels based in boronic-ester systems has been recently reported [47]. The nanogels were prepared by reaction between HA-PBA and dextran (Dex) modified with fructose (Fru) or maltose (Mal) moieties. It was found that nanogels prepared from HA-PBA/Dex-Mal presented quicker instability than HA-PBA/Dex-Fru, which was attributed to a faster cinematic exchange in the boronate-ester bond. The nanogels also showed pH responsiveness: particle formation at pH above 7 and dissolution at lower pH, being this change reversible at pH above 7 (the nanoparticles re-form again).

Boronic-ester-based hydrogels are biocompatible, display self-healing properties and thus possess a wide range of purposes in the biomedical field. They have been used as injectable materials with H_2_O_2_/Reactive Oxygen Species (ROS) responsive properties. Moreover, these systems display applications in tissue engineering due to their ability to encapsulate different cell lines, such as neural progenitor cells (NPCs) and mouse embryonic fibroblasts (MEFs), and to behave as bio-inks for 3D printing and bioprinting [36,45]. Finally, hyaluronic acid hydrogels cross-linked with boronic acid derivatives can be employed as drug delivery systems for the release of active substances such as dihydrocaffeic acid (DHCA), which prevents the photoaging of the skin [43].

### 2.2. Schiff-Base Formation

**Figure 4:**
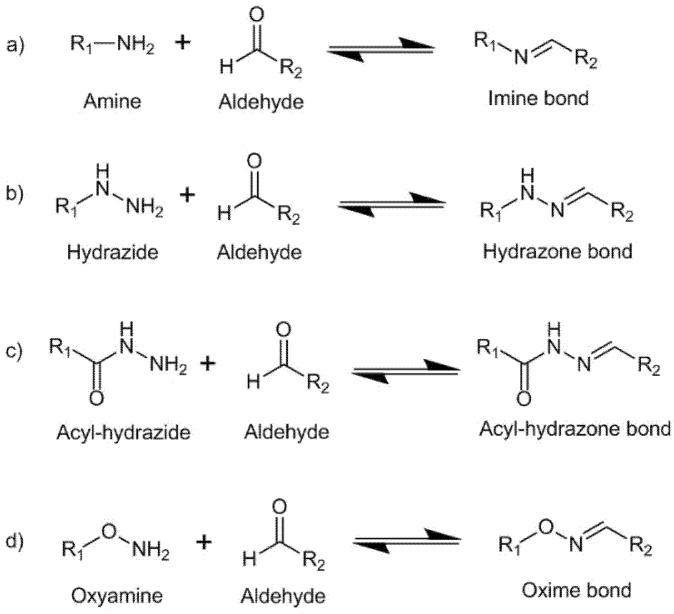
Reversible Schiff-base formation, (**a**) imine, (**b**) hydrazone, (**c**) acyl-hydrazone, and (**d**) oxime bonds through reactions between aldehyde groups and primary amine, hydrazide, acyl-hydrazide, or oxyamine groups, respectively.

Imine bonds, hydrazone bonds, acylhydrazone bonds, and oxime bonds can be formed by Schiff-base reaction between an active carbonyl group and various nucleophilic amine groups, reaction schemes are shown in Figure 4. These bonds can present dynamic behavior under mild conditions, endowing the material with reversibility and self-healing properties which allow their injectability [48,49].

A common way to functionalize HA with active carbonyl groups is through the oxidation of vicinal hydroxyl groups with sodium metaperiodate (NaIO_4_). The oxidation reaction breaks the C-C bond and produces two aldehyde groups at the oxidized carbons (two vicinal aldehyde groups). Then, the oxidized HA (OHA) can be crosslinked by Schiff-base reactions between aldehyde and amine groups [50], see Figure 4a. Chitosan (CS), a natural polysaccharide containing amine groups with good biocompatibility and biodegradability, is an excellent candidate to be employed along with OHA in hydrogel formation under physiological conditions. Carboxyethyl–chitosan (CEC) biopolymer reacts with OHA to form dynamic hydrogels under physiological conditions. The hydrogels presented self-healing properties allowing for complete healing within 3 min giving the injectable capacity to the system. In addition, swelling dependence with pH was found in the hydrogels which enabled their employment as matrixes for controlled drug delivery. At acidic pH, the hydrogels showed lower swelling increasing the drug delivery but also at these acidic conditions the protonation of amino groups broke the dynamic bond improving the interconnectivity of the network. The hydrogels showed excellent biocompatibility and biodegradability [51,52]. Injectable hydrogels have also been obtained through the reaction between OHA and Glycol Chitosan (GC) under mild conditions [53]. The authors studied the influence of the degree of oxidation of HA, the mixing ratio of OHA/GC, and the final polymer concentration on the mechanical properties of the hydrogels. Recently, Graphene oxide (GO) was added to OHA/GC hydrogels to improve their osteogenic functionalities [54].

In addition, the introduction of adipic acid dihydrazide (ADH) to the OHA/GC hydrogel system at physiological conditions affords acylhydrazone bonds from the reaction between OHA and ADH [55] (Figure 4b). This way, the exchange kinetics of the dynamic bonds is altered, the stiffness of the hydrogel is reduced, and as a consequence self-healing properties are generated into the network. Hydrogels based on HA-ADH/OHA were also obtained with good cytocompatibility and hemocompatibility [56,57]. Hyaluronic acid hydrogels formed from OHA, through oxidation and breakage of the sugar ring, show less stability due to the tendency of the ring-opened structure to hydrolyze [58]. Hence, an alternative strategy to functionalize HA with active carbonyl groups proceeds via pre-modification and subsequent oxidation of HA. Thus, HA was pre-modified with -3-amino-1,2-propanediol followed by oxidation to yield a mono-aldehyde HA (HA-mCOH). The aldehyde groups were then coupled with gelatin containing a hydrazide group under mild conditions. The stability of hydrogels formed from HA-mCOH was higher when compared to that exhibited by hydrogels formed from hyaluronic acid oxidized through breakage of the sugar ring [59].

HA hydrogels crosslinked through oxime bonds can be formed by reaction between aldehyde or ketone groups with oxyamine groups (Figure 4d). The kinetics of the reaction at physiological conditions can be modified and accelerated through the addition of different salts, which can be considered as bio-friendly and non-toxic catalysts [60]. Injectable HA hydrogels based on oxime chemistry were recently obtained through the reaction between a polyethylene glycol (PEG) functionalized with oxyamine groups and a HA modified with aldehyde and ketone groups. The hydrogels showed tunable gelation times ranging from 15 min to 0,4 min by increasing aldehyde concentration, which enabled their injectability. The hydrogels showed cytocompatibility with retinal cells and a controlled swelling being able to maintain the ocular pressure in vivo (rabbit assays) over 56 days maintaining a healthy functional retina [61].

Hyaluronic acid hydrogels produced by Schiff-base reactions do have a broad scope of uses in biomedicine due to their biocompatibility and self-healing capabilities. These systems display applications in tissue engineering because of their ability to encapsulate various cell lines, such as chondrocytes and ATDC5 cells that promote cartilage regeneration [53,55]. Schiff-base grounded hyaluronic acid hydrogels also induce angiogenesis processes [59], enhance the osteogenic functionalities and mechanical properties of bone tissue [54], and may be used as bioinks for fabricating cell-laden structures using a 3D printer [55] and as vitreous substitutes for ophthalmological applications [61]. These types of hydrogels have been employed as drug delivery systems for pH-mediated release of the anticancer drug Doxorubicin (Dox) [51]. Most of these materials are injectable [51,53,54,55,59] which facilitates the dispensing of the hydrogel and thus the final application of the hydrogel (delivery of active substances or scaffolds for tissue engineering among others).

### 2.3. Thiol Chemistry

**Figure 5:**
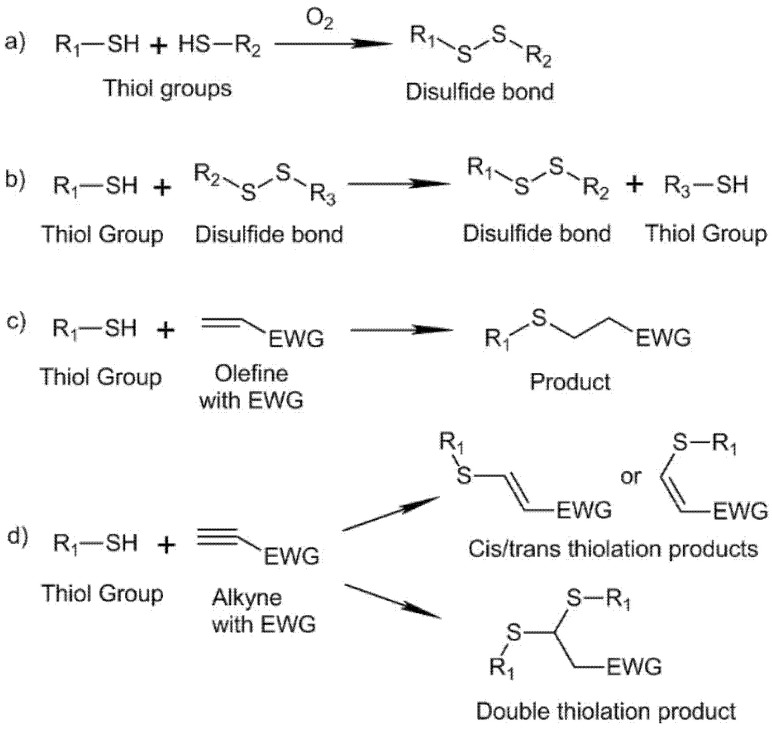
Thiol Chemistry: (**a**) Disulfide formation through oxidation reaction, (**b**) disulfide-exchange reaction, (**c**) Michael addition reaction, and (**d**) thiol–yne addition reaction.

Polymers displaying naturally occurring amino and thiols groups are of great interest because in the presence of biological components, are able to achieve chemo-selectivity towards them and to induce crosslinking reactions. Compared to the amino group, the thiol group (S-H) occurs at a lower abundance in naturally existing molecules. Moreover, at the physiological pH, the nucleophilicity of thiol is 1000 times stronger than the ionized amino group and therefore bears relatively higher chemoselectivity [18,19]. The thiol group can undergo several reactions; in the present work, we focus our efforts on those that can be carried out in physiological conditions and that require no catalysis to occur: disulfide formation/exchange reactions, Michael addition type reactions, and thiol–yne addition reactions. Reaction schemes are collected in Figure 5.

The thiol group can react through disulfide formation/exchange reactions. The S-H group participates in the formation of disulfide bonds (S–S) that are essential to the tertiary structures of proteins. This functionality is also present in the active sites of many enzymes. Moreover, disulfide formation can be employed to form HA hydrogels under physiological conditions. The oxidation reaction involves the thiol deprotonation and subsequent reaction with oxygen, so the reaction kinetics is highly influenced by the pKa of the thiol and their deprotonation degree under physiological conditions [62,63,64] (Figure 5a). Introducing an electron-withdrawing group (EWG) in the thiol group can modify and lower the pKa, increasing its reactivity [65]. Disulfide-based hyaluronic acid can be promoted using different oxidants, such as dimethyl sulfoxide (DMSO) [66] or iodine [67]. Parallel to disulfide formation, the thiol group can also undergo a disulfide exchange reaction, which endows the system with self-healing capacity. Disulfide exchange occurs between a deprotonated thiol (nucleophile) that reacts with a disulfide under basic conditions (pH 7–9) and the system could deactivate by oxidation of thiol groups (O_2_) or protonation (acid conditions) [68,69] (Figure 5b). Furthermore, some assays have evaluated the induction of disulfide exchange reactions into a disulfide system with glutathione (GSH, a small molecule produced by cells). A reduction in the storage modulus was found after the incorporation of GSH into the network, and after incubation in PBS the modulus showed partial recovery, proving the reversibility of the system, i.e., breakage and reforming of the disulfide bonds [70].

A second reaction for the formation of HA hydrogels involving thiol groups is the addition of thiol groups to alkenes via Michael addition. The reaction is a nucleophilic addition of a thiol group (Michael donor) to an olefine with an EWG (Michael acceptor) under basic conditions, with the thiol group in the anion form (Figure 5c). Moreover, due to the inherent electron density of the S atom, thiol-Michael addition could co-exist in mild aqueous conditions with photopolymerization, which has been utilized to design step cross-linkable hydrogels. When the light intensity is low and photopolymerization occurs in an alkaline solution, the rates for photopolymerization and thiol-Michael addition are comparable [71]. Polyethylene glycol (PEG) containing diacrylate/methacrylate (PEGDA/PEGDMA) groups are traditionally employed to crosslink thiolated HA for the formation of hydrogels through Michael addition under physiological conditions (pH 7,4) [72,73]. In this type of hydrogel formation, a rapid Michael addition reaction (rapid gelation) co-exists with the slow disulfide formation through the oxidation of the thiol group (prolonged crosslinking). To minimize the disulfide formation, a molecule with a mono-functionality can be added to the system for thiol end-capping and avoid oxidation [74,75,76]. For thiolated HA-based hydrogels obtained through the employment of PEGDA as a crosslinker, gelation times of 9 min were obtained [77].

Besides the employment of acrylate and methacrylate groups as Michael acceptor groups, Michael addition reactions may also involve thiol and maleimide groups. HA-based hydrogels were obtained through a dual crosslinking mechanism. As a first step, HA was modified either with maleimide or thiol groups. Then, after mixing the modified HA fractions, a rapid Michael addition between both groups occurred by adjusting the pH to physiological conditions. After the initial gelification, a disulfide formation between the thiol groups happened [78,79]. Michael addition may also proceed under physiological conditions between a vinyl sulfone and a thiol group. This way, HA hydrogels have been produced through the reaction of thiolated HA and PEG-vinyl sulfone under physiological conditions with gelation times ranging from 14 min to less than 1 min [80,81].

A third strategy for HA crosslinking involving thiol groups is the thiol–yne addition reaction. Thiol-yne coupling can occur through radical or nucleophile pathways. In this review, we focus on the nucleophilic mediated thiol-yne addition reaction, highly suitable for hydrogel synthesis because of its efficiency and rapid nature [82] (Figure 5d). HA-SH may react with PEG-yne derivatives displaying different architectures to afford hydrogels in a few minutes. It provides a straightforward and suitable approach, with few synthetic steps, to prepare robust HA click-hydrogels, which intrinsically possess cell adhesion capability and display a superior mechanical performance [83].

Thiol chemistry-mediated hyaluronic acid hydrogels are biocompatible [62,63,72,77,79,83,84,85] and show self-healing and bio-adhesive properties [85]. They have been employed as injectable materials [77,78,83,84] which facilitate the application in the human body for tissue engineering purposes These types of hyaluronic acid hydrogels are able to encapsulate cells such as L-929 murine fibroblasts that remained viable and proliferated in vitro [62]. S. Bian et al. encapsulated chondrocytes and L929 cells in hydrogels that were able to proliferate and aggregate forming an extracellular matrix (ECM) [63]. Cartilage-derived progenitor cells (CPCs) were encapsulated to overcome cell delivery drawbacks [84]. Encapsulated CPCs retained a high level of cell viability and proliferation capabilities. Moreover, encapsulated CPCs remained functional as extracellular matrix (ECM) secretion was enhanced under chondrogenic conditions, and the inflammation gene expression was downregulated which indicated the anti-inflammatory ability of encapsulated CPCs. Human mesenchymal stem cells (hMSC) as Y201 hTERT-immortalized human clonal MSCs57 [83] and Passage 3 human MSCs [79] were also encapsulated in hydrogel networks for chondrogenic applications. T31 tracheal scar fibroblasts were encapsulated in hydrogels and implanted subcutaneously in the flanks of nude mice. Immunohistochemistry indicated that the encapsulated cells retained the fibroblast phenotype and secreted extracellular matrix in vivo [77]. Many of these encapsulation systems have been used as 3D scaffolds for tissue engineering [63,83]. Other applications in tissue engineering include the generation of biocompatible and biodegradable substrates for in vitro cell culture [64], the production of hydrogel matrixes for wound healing [72], and hemostatic applications [85].

### 2.4. Cycloaddition Reactions

#### 2.4.1. Azide-Alkyne Cycloaddition Reaction

**Figure 6:**
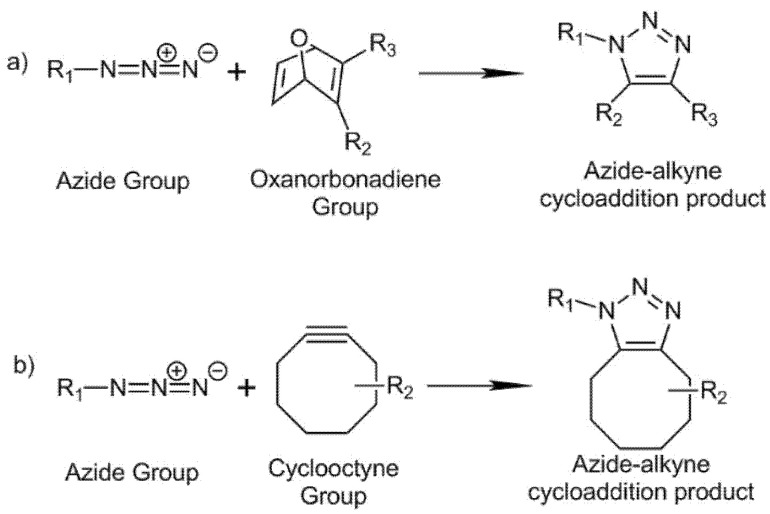
Strain-promoted azide–alkyne cycloaddition (SPAAC) reaction between azide and (**a**) oxanorbonadiene and (**b**) cyclooctune groups.

Traditionally, azide–alkyne cycloaddition “click” reaction has been catalyzed with copper (I) to form a triazole ring. The reaction is characterized by its high selectivity, rate, yield, and can proceed under ambient conditions. However, the use of metals in the reaction limits the employment of these reactions for the development of materials for biomedical applications [86,87]. In the present decade, strain-promoted azide–alkyne cycloaddition (SPAAC) “click” reaction has gained importance due to its metal-free click chemistry development needing no catalyst at physiological conditions [88]. This reaction can be applied for “in situ” hydrogel formation using an alkyne functional group oxanorbonadiene or cyclooctyne structures [89,90,91], see Figure 6.

Azide-modified HA (HA-AA) reacted with oxanorbonadiene-modified CS (CS-OB) to afford HA hydrogels after 23 min (ratio AA:OB = 1:1, 2 wt%). The hydrogel showed cell encapsulation capability and biocompatibility. In addition, in vivo assays showed the viability of this material for in situ gelation when injected in mice [89]. The modification of HA with cyclooctyne groups has also been employed as a strategy for crosslinking of HA-based hydrogels at physiological conditions [90]. Cyclooctyne–HA reacted with azide–PEG with gelation times of 5 min (ratio azide/cyclooctyne 2:1, 5 wt%). The hydrogels showed low toxicity and good biocompatibility. Encapsulation of cells within the HA-based hydrogels increased gelation time to 10 min. Cell proliferation was not affected in encapsulated cells as compared with non-encapsulated cells employed as control experiments.

Hyaluronic acid hydrogels generated by azide-alkyne cycloaddition reaction are biocompatible and show a wide range of purposes in tissue engineering. These materials are able to encapsulate in vitro human adipose-derived stem cells (ASCs) [89], COS-7 fibroblast-like cell lines derived from monkey kidney tissue [90], and chondrocytes [91] which demonstrate their potential as cell scaffolds for 3D cell culture. Furthermore, these hydrogels are injectable, which simplifies their application in vivo for adipose tissue scaffolding [89], regeneration of cartilaginous tissue [91], and dermal filling in plastic surgery [90].

#### 2.4.2. Diels–Alder Formation

**Figure 7:**
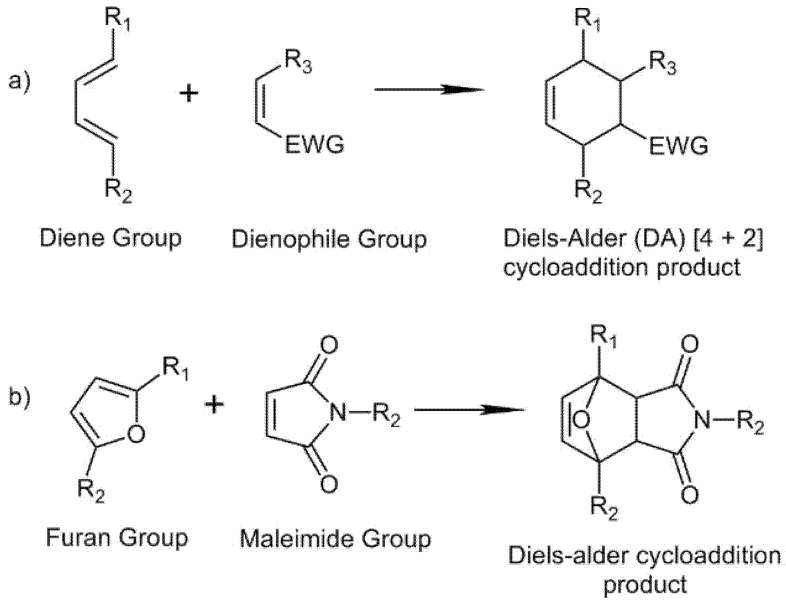
Diels–Alder formation: (**a**) Diels–Alder general [4 + 2] cycloaddition reaction. (**b**) DA cycloaddition reaction between furan and maleimide groups.

Covalent crosslinked hyaluronic acid hydrogels can be obtained through Diels–Alder (DA) [4 + 2] cycloaddition between an electron-deficient dienophile and an electron-rich conjugated diene (Figure 7a). DA cycloaddition can occur under mild conditions and exhibits a dynamic temperature behavior [92,93]. An example of HA hydrogels obtained through the DA reaction is those formed between furan-modified HA which acts as a diene and PEG-maleimide which acts as a dienophile (Figure 7b). The hydrogels formation is performed under acidic pH conditions in MES buffer (pH 5.5) [94,95,96]. To accelerate the DA reaction at physiological conditions, a more electron-rich furan can be achieved through the introduction of a methyl group in its structure [97].

**Figure 8:**
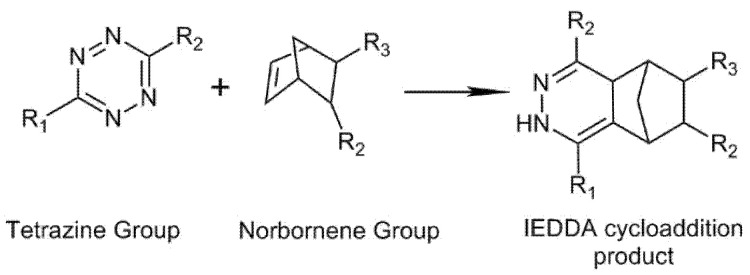
Inverse electron demand Diels–Alder (IEDDA) cycloaddition reaction between tetrazine and norbornene groups.

HA hydrogels are also formed by inverse electron demand Diels–Alder (IEDDA) cycloaddition reaction using tetrazine groups as the diene to react with alkenes or alkynes under physiological conditions (see Figure 8). The material formulations can be “in situ” injected into the area to be treated and rapidly form the crosslinked network, due to the “click” properties of the reaction and do not require an external reagent [98,99,100,101,102]. HA-tetrazine (Tet) can react with PEG-norbornene to form DA hydrogels. The gelation time was found to be dependent on the modification degree of HA-tetrazine, the temperature, and the concentration of the precursors reaching gelation times lower than 1 min. In addition, the hydrogel showed protein encapsulation capacity and a subsequent release for several weeks [101].

Dual-crosslinked hydrogels can be produced by mixing two mechanisms of reaction independently activated which allows the system to fulfill complex needs and opens new applications. As an example, the combination of DA cycloaddition and condensation reactions results in robust hydrogels for potential cartilage regeneration [103]. As a first step, a quick dynamic condensation reaction between phenylboronic acid (PBA) and dopamine groups introduced onto the HA backbone gives rise to hydrogels with shear-thinning properties, able to be injected. Then, a second slower DA reaction occurs endowing the material with higher mechanical properties.

**Figure 9:**
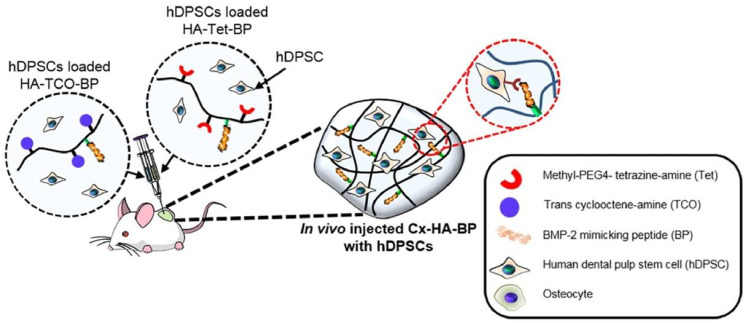
The figure shows that BMP-2 mimicking peptide (BP) loaded within an injectable ‘Diels Alder’ hyaluronic acid hydrogel induces the osteogenic differentiation of human dental pulp stem cells (hDPSC). Adapted with permission from [100].

Diels–Alder-based hyaluronic acid hydrogels do have an extensive sort of use in biomedicine due to their biocompatibility and injectability [98,99,100,103]. These systems have been applied in tissue engineering due to their ability to encapsulate cells. Breast cancer cell lines (BT474, MCF7, and T47D) and glioma neural stem cell lines (G523, G411) were successfully encapsulated for 3D cell culturing [97]. Encapsulated chondrogenic ATDC-5 cells successfully proliferated in a hydrogel environment which proves the potential applications in the field of cartilage regeneration [103]. Diels–Alder-based hydrogels not only supported cell encapsulation but were also able to vehiculize active substances for combined tissue engineering and drug delivery applications. In this regard, methotrexate-loaded hydrogels increased cartilage thickness, chondrocyte generation, and induced new bone formation in rheumatoid arthritis-affected body regions [99]. Dexamethasone (Dexa)-loaded microspheres mixed with cross-linked hyaluronic acid hydrogel displayed a retarded release of the Dexa in vitro and in vivo and represent a suitable method for the treatment of inflammatory and autoimmune disorders, such as rheumatoid arthritis [98]. Finally, bone morphogenetic protein-2 (BMP-2) mimetic peptide (BP) was loaded and released from an injectable hyaluronic acid hydrogel obtained through a Diels–Alder reaction between modified HA-tetrazine and HA- trans-cyclooctene (TCO). This system induced the osteogenic differentiation of hDPSCs which supports the use of this material for bone tissue engineering applications (Figure 9) [100].

Table 1 summarizes the crosslinking reactions in this review with an indication of the functional groups involved in the crosslinking, the properties of the resulting hydrogels, and their biomedical applications.

## 3. Thermoreversible Gelation of HA Hydrogels at Physiological Temperature

Thermo-sensitive hydrogels, which undergo in situ sol-gel transition at physiological temperature, without chemical or enzymatic modification, are widely studied as biomaterials to be employed in tissue engineering and long-term controlled drug release [104] Among them, physical thermoreversible hydrogels obtained through noncovalent interactions (hydrogen bonding, ion crosslinking, or hydrophobic interactions, among others) have attracted considerable attention over the years because of the mild conditions required for the formation of crosslinking points [105].

Poly(N-isopropylacrylamide) (PNIPAm) is one of the most investigated thermosensitive polymers. It shows a reversible sol-gel phase transition induced by a hydrophobically induced reorganization/aggregation that occurs at its low critical solution temperature (LCST) at ~32 °C. Such LCST can be modulated to get closer to physiological temperature. Thermosensitive hyaluronic hydrogels can be obtained through the incorporation of PNIPAm into the backbone of hyaluronic acid (i.e., grafting). For that, PNIPAm was end-capped with a carboxylic acid group or amine groups and then grafted to HA carboxylic acid groups through carbodiimide coupling chemistry. It is generally found that grafted hyaluronic acid with PNiPAAm showed a similar LCST to PNiPAAm. In addition, as the amount of PNiPAAm grafted to HA increased the copolymers solutions showed a narrower temperature range for LCST transition. Such materials have been investigated as drug delivery materials [106,107]. Another approach to provide HA with thermoresponsive properties is the formation of semi-interpenetrating polymer networks constituted of PNiPAAm and hyaluronic acid through polymerization of the monomer N-isopropylacrylamide (NIPA) in aqueous solutions of hyaluronic acid [108].

Other thermosensitive systems widely studied for biomedical applications are amphiphilic polyethylene glycol (PEG)-based copolymers such as Pluronics which is a PEG–polyester-based triblock consisting of hydrophilic poly (ethylene oxide) (PEO) and hydrophobic poly (propylene oxide) (PPO) blocks arranged in a basic A-B-A tri-block structure. Physical mixing of high-molecular-weight HA with Pluronics results in thermosensitive hydrogels with enhanced mechanical properties and sustained drug release behavior, thus avoiding burst drug release reported for Pluronic copolymer hydrogels employed as biomaterials for intra-articular injection [109]. Another approach consists of chemical modification of HA and Pluronics with functional groups liable to in situ crosslinking. Pluronics can be end-capped with thiol groups and then react with HA conjugated with dopamine through a Michael-type addition. Interestingly, even if these HA-DN/Pluronics gels were chemically cross-linked, they exhibited thermally reversible sol-gel transition which was found to appear at temperatures lower than the corresponding to Pluronics hydrogels [110]. Grafting of Pluronics to HA results in copolymers being able to gelify with temperature. The Pluronics grafting percent influences the sol-gel temperature, i.e., at the highest Pluronics grafting percent, (86.4%) the copolymer showed a slightly higher gelation temperature compared to the unmodified Pluronics at all concentrations [111].

### Combination with Natural Polymers

**Figure 10:**
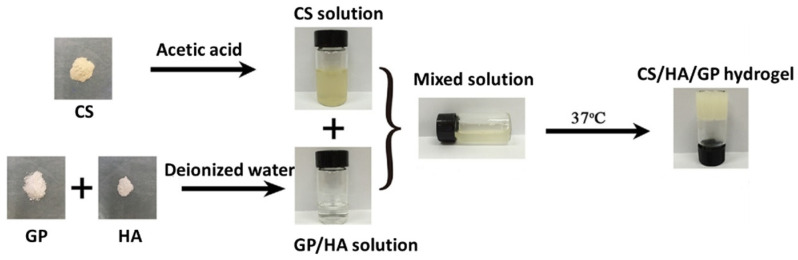
Schematic representation of the experimental procedure for the preparation of thermosensitive hyaluronic (HA) hydrogels prepared through blending of solutions of chitosan (CS) and β-sodium glycerophosphate (GP) at 37 °C. Taken with permission from [114].

Hydrogels obtained through a combination of hyaluronic acid with other natural polymers, mainly polysaccharides and proteins, exhibit intrinsic properties of biocompatibility and biodegradability that are very useful in the biomedical field. Thermosensitive chitosan gels were first reported by Chenite et al. and can be obtained through cross-linking with β-sodium glycerophosphate, sol-gel transition occurring at physiological temperature and pH [112]. The combination of chitosan/β-sodium glycerophosphate gels with polyvinyl alcohol hydrophobically modified gives rise to hydrogels with enhanced mechanical properties [113]. The mixing of chitosan and hyaluronic acid followed by crosslinking with β-sodium glycerophosphate (see Figure 10) has also been employed for the preparation of injectable hydrogels that can be applied for the release of antitumoral drugs [114].

Chitosan derivatives such as hydroxypropyl chitin (HPCH) have also been combined with HA to obtain thermosensitive hydrogels. Hydroxypropyl chitin can form hydrogels at 37 °C at low concentrations (~2 wt%). The mixing of HPCH with hyaluronic acid gives rise to polymer hydrogels able to stabilize negatively charged compounds for drug release applications [115]. A similar approach has been used for the development of thermo-sensitive HA physical hydrogels with anti-adhesion properties as a strategy to alleviate surgery-related adhesions. To that aim, methylcellulose has been mixed with HA. Methylcellulose (MC) is a typical temperature-responsive water-soluble polymer that can gel at 37 °C in the presence of salts [116].

It is reported that aqueous solutions of gelatin, a natural polymer obtained from the partial denaturation of collagen, are able to form physical hydrogels upon cooling below room temperature [117]. However, the employment of gelatin hydrogels for biomedical applications is limited since the melting temperature of gelatin hydrogels is ~32 °C and thus, under physiological conditions, gelatin gels are unstable and present poor mechanical properties. Very recently, an approach based on natural polyelectrolyte complexes coatings has been developed in order to endow gelatin with dimensional stability at a physiological temperature [118]. Blends of hyaluronic acid with gelatin give rise to reinforced hydrogels that can act as biomimetic hydrogels formed by a protein and a polysaccharide being similar to extracellular matrix structure [119].

A combination of hyaluronic acid with natural polymers can be also achieved by crosslinking with genipin. Genipin is a naturally derived cross-linking agent with negligible cytotoxicity that has been extensively used for the formation of hydrogels from natural polymers, most prominently for the crosslinking of chitosan [120]. Genipin cross-links polymers containing primary amine groups. In these regards, injectable hydrogels were obtained through the combination of collagen, chitosan, and lysine-modified hyaluronic acid crosslinked with genipin [121]. The mechanical properties of the resulting hydrogels were influenced by the degree of modification of the HA and the genipin concentration. The hydrogels were biocompatible and presented antibacterial activity against *Escherichia coli*. A similar approach was employed to form gelatin/HA crosslinked hydrogels involving genipin as the crosslinker [122]. Researchers also used HA to form polyelectrolyte complexes (PECs) based on chitosan/alginate/hyaluronic acid or collagen/chitosan/hyaluronic acid under mild conditions. The amine moieties present in chitosan or collagen were crosslinked [123,124].

## 4. Conclusions

Research on hyaluronic acid hydrogels for the development of biomedical applications currently attracts great interest due to the versatility of this biopolymer to be chemically functionalized to meet the characteristics required for a wide range of biomedical applications from scaffolds for tissue engineering to matrixes for controlled drug release. Currently, many of the hyaluronic acid hydrogel formulations, commercialized mostly as injectable biomaterials, are obtained through reaction conditions that are not compatible with cell culture which hinders some of their bio-applications. Nowadays, click chemistry constitutes a powerful toolkit for the development of novel formulations of hyaluronic-based hydrogels through bioortoghonal reactions that are carried out at physiological pH and temperature as those reviewed in the current manuscript (Schiff-base formation, thiol chemistry, azide-alkyne, or Diels–Alder cycloaddition). In addition, the setup of novel strategies to obtain dynamic covalent hydrogels based on hyaluronic acid, of which the boronic–ester formation is an example, opens new perspectives for emergent bio-applications. Dynamic covalent crosslinked hydrogels present injectability, self-healing properties, and stimuli responsiveness and maintain properties and the stability that characterize covalent systems. Current trends in the development of hyaluronic acid hydrogels exploit the formation of dual crosslinked networks having different crosslinking kinetics that allows tuning their rheological properties for the envisaged application (i.e., tuning gelation time for shear-thinning and gel formation in 3D printing or mimic of the rheological properties of complex biological tissues).

## Figures and Tables

**Table 1:** Crosslinking reactions at physiological conditions.

Cross-Linking	Complementary Groups	HA Chemical Modification	Biomedical Applications
**Boronic–ester formation**	Boronic acid + amine/hydroxyl [36,42,43,45,47]	Amidation [36,42,43,45,47];	Injectable [36,45]; biomaterial inks [36]; tissue engineering [36]: bone; ROS-responsive properties [36]; drug release [43]
**Schiff base formation**	Amine + Aldehyde [51,53,54,55,59]	Oxidation [51,53,54,55]; Amidation [59,61];	Injectable [51,53,54,55,59,61]; tissue engineering [59]: bone [54], cartilage [53,55]; Bioprinting [55]; pH responsive property [51]; drug release [51]; vitreous substitute [61]
	Dihydrazide + Aldehyde + [56,59]		
	Oxyamine + aldehyde/Ketone [61]		
**Thiol Chemistry**			
**Disulfide formation/exchange**	Thiol-thiol [62,63,64,74,75]	Amidation [62,63,64,74]	Tissue engineering [62,63,64]
**Michael addition**	Thiol + (metha)/acrylate [72,73,74,75,76,77,84]	Amidation [72,74,77,78,79,81,85]; ether formation [80]	Injectable [77,78,81,84]; tissue engineering [72,73,77,84]: cartilage [79,81]; cell encapsulation [84]; wound healing [72]; hemostasis [85]
	Thiol + Melaimide [78,79]		
	Thiol + Vinyl-sulfone [80,81]		
	Thiol + Catechol [85]		
**Thiol-yne coupling**	Thiol/yne [83]	Amidation [83]	Injectable [83]; cell encapsulation [83]; tissue engineering: cartilage [83]
**Cycloaddition reaction**			
**Azide–alkyne cycloaddition reaction**	Azide + Oxanorbornadiene [89]	Amidation [89,90,91]	Injectable [89,90,91]; tissue engineering [89]; cell encapsulation [90,91]
	Azide + Cyclooctyne [90,91]		
**Diels–Alder formation**	Furan + Maleimide [97,103]	Amidation [97,98,99,100,101,102,103]	Injectable [98,99,100,103]; tissue engineering [99,103]: bone [100]; rheumatoid arthritis [99]; cell encapsulation [97]; protein encapsulation [101]; drug release [98,99]
	Tetrazine + Trans-cyclooctene [98,99,100]		
	Tetrazine + norbornene [101,102]		

## Data Availability

Not applicable.

## Abbreviations

Abbreviation	Name
ADH	Adipic acid dihydrazide
ASCs	Adipose-derived stem cells
pKa	Acid dissociation constant
HA-AA	Azide-modified HA
BP	BMP-2 mimicking peptide
CEC	Carboxyethyl-chitosan
CPCs	Cartilage-derived progenitor cells
CS	Chitosan
Dex	Dextran
Dexa	Dexamethasone
DA	Diels-Alder
Dox	Doxorubicin
DHCA	Dihydrocaffeic acid
DVS	Divinyl sulfone
ECM	Extracellular matrix
EWG	Electron withdrawing group
Fru	Fructose
GP	Glycerophosphate
GC	Glycol Chitosan
GLU	Gluconamide
HA	Hyaluronic acid
HA-tet	HA-tetrazine
HA-TCO	HA- trans-cyclooctene
hMSC	Human mesenchymal stem cells
BMP-2	Human bone morphogenetic protein 2
HPCH	Hydroxypropyl chitin
IEDDA	Inverse electron demand Diels-Alder
G‘‘	Loss Modulus
LCST	Low critical solution temperature
Mal	Maltose
HA-mCOH	Mono-aldehyde HA
NIPA	N-isopropylacrylamide
CS-OB	Oxanorbonadiene-modified CS
OHA	Oxidized Hyaluronic acid
PBA	Phenylboronic acid
PECs	Polyelectrolyte complexes
PEG	Polyethylene glycol
PEGDA	Polyethylene glycol di-acrylate
PEGDMA	Polyethylene glycol di-methacrylate
PEO	Poly (ethylene oxide)
PNIPAm	Poly (N-isopropylacrylamide)
PPO	Poly (propylene oxide)
QbD	Quality by design approach
ROS	Reactive Oxygen Species
NaOH	Sodium hydroxide
NaIO4	sodium periodate
T	Temperature
SH	Thiol group
3D	Three-dimensional
G’	Storage Modulus
SPAAC	Strain-promoted azide–alkyne cycloaddition
UV	Ultraviolet
